# Interkingdom Biofilms in Chronic Wounds: The Collaboration of *Candida albicans* and *Staphylococcus aureus* Against Conventional Wound Antiseptics in a Wound-like Leucocyte-Rich Human Plasma Biofilm Model (lhBIOM)

**DOI:** 10.3390/antibiotics15080739

**Published:** 2026-07-31

**Authors:** Mandy Dittmer, Sophie C. Liegenfeld, Nicolas Krueger, Maria Geffken, Arianna Delle Coste, Caroline Schoeller, Ifey Alio, Wolfgang R. Streit, Ewa K. Stuermer

**Affiliations:** 1 Department of Vascular Medicine, Translational Wound Research, University Heart Center, University Medical Center Hamburg-Eppendorf, 20246 Hamburg, Germany; s.liegenfeld@uke.de (S.C.L.); ni.krueger@uke.de (N.K.); caroline.schoeller@stud.uke.uni-hamburg.de (C.S.); 2 Institute for Transfusion Medicine, University Medical Center Hamburg-Eppendorf, 20251 Hamburg, Germany; maria.geffken@uke.de; 3 Department of Oral and Maxillofacial Surgery, University Medical Center Hamburg-Eppendorf, 20246 Hamburg, Germany; a.dellecoste@uke.de; 4 Department of Microbiology and Biotechnology, University Hamburg, 20148 Hamburg, Germany; ifey.alio@uni-hamburg.de (I.A.); wolfgang.streit@uni-hamburg.de (W.R.S.)

**Keywords:** *S. aureus*, *C. albicans*, biofilm, interkingdom biofilm, chronic wound, antiseptics, antimicrobials

## Abstract

Background: Chronic wounds are frequently associated with biofilms, in which not only bacterial but also fungal pathogens can impair wound healing. Among the most relevant opportunistic pathogens is *Staphylococcus aureus*; together with *Candida albicans*, both are part of the human skin microbiome but can also colonize chronic wounds. Interkingdom biofilms formed by these microorganisms have been shown to exacerbate the course of diseases compared to infections caused by either species alone. Methods: To address the limited number of studies examining fungal–bacterial interactions in wound environments, leucocyte-rich human plasma biofilm models (lhBIOMs) inoculated with *S. aureus* and *C. albicans* were prepared. The efficacy of the commonly used clinical antiseptics octenidine dihydrochloride/phenoxyethanol (OCT/PE) and polyhexamethylene biguanide (PHMB) was examined using the quantitative suspension method (QSM). In addition, spatial distribution and morphology of the microorganisms within this biofilm model were analyzed by confocal laser scanning microscopy (CLSM). Results: In this study, the presence of *S. aureus* triggered an increase in the formation of filamentation of *C. albicans* in contrast to the single-species biofilm. In addition, treatment with the tested antimicrobial agents was effective against *C. albicans* after repetitive applications and showed a clear reduction against *S. aureus*. Furthermore, the quantitative analysis of the co-culture revealed increased growth of *S. aureus* in the control culture compared to the single-species model. Conclusions: These findings highlight the pathogenic relevance of interkingdom biofilms in chronic wounds and emphasize the importance of effective species-independent antimicrobial treatment strategies.

## 1. Introduction

Chronic wounds represent a major healthcare challenge in aging populations, as they are associated with pain, social isolation, physical limitations, and even limb amputation. By definition, a wound is considered chronic if it has not healed within 8 weeks, especially if there is an underlying disease such as peripheral arterial disease (PAD) or diabetes, which can lead to hard-to-heal wounds [1].

In Europe, chronic wounds have an estimated prevalence of approximately 2.5%. In Germany alone, an estimated 1 to 4 million people are affected [2]. Chronic wounds provide favorable conditions for various microorganisms; consequently, more than 75% of all chronic wounds are associated with biofilms [3,4,5].

Unfortunately, the biofilm and its matrix protect the embedded microorganisms from the (already impaired) host immune defenses and increase the tolerance and resistance to antimicrobial treatment [6,7,8].

An example of a biofilm-forming wound pathogen is *Staphylococcus aureus* (*S. aureus*). This Gram-positive bacterium is part of the healthy skin microbiome but also one of the five most prevalent bacteria in acute and chronic wounds and one of the most widespread pathogens [5,9,10].

Several studies have investigated wound-associated biofilms and their biofilm-forming bacteria, such as *S. aureus*, which is one of the predominant pathogens associated with chronic wound infections [4,11,12,13]. However, bacteria should not be considered the only factor influencing the pathology of an infected chronic wound. Fungal and yeast infections may aggravate tissue damage and delay healing [4,11]. Nevertheless, in clinical wound routines, mycoses are widely underestimated [4,11,12]. Previous studies have reported fungal colonization in 23–27% of chronic wounds, with prevalence rates of up to 40% in diabetic wounds, with *Candida* spp. being the most common fungal species found in chronic wounds [4,14].

One known opportunistic pathogen species in this genus is *Candida albicans*. This yeast is part of our human skin microbiome. It can be found in and on 30–70% of healthy humans (e.g., skin, genital mucosa, and intestinal mucosa), but it is also responsible for about 70% of fungal infections worldwide [15,16,17]. A typical characteristic and important virulence factor of *C. albicans* is the various reversible morphological states in which this yeast can appear [17,18]. In our skin microbiome, *C. albicans* occurs primarily in the blastospore form, in which it can reproduce asexually by budding [17]. Furthermore, this fungus is capable of forming filamentous structures (pseudohyphae and hyphae). Here, pseudohyphae are a transitional form between cellular and hyphal morphologies and can assume a variety of growth forms. This morphological state manifests as elongated cells with constrictions at the septa, resulting from buds that expand, form septa, and remain attached to the mother cells [17,19]. Initially, nuclear division, similar to yeast cells, occurs across the mother bud neck. In contrast, hyphae formation starts with the migration of the nucleus out of the mother cell, where it divides within the germ tube. The hyphae state shows less branching than the pseudohyphae state [19]. Due to the invasive hyphae form, cells enter host tissue by active penetration and induced endocytosis [20]. Besides that, candidalysin, a toxin produced by *Candida* hyphae, causes cellular damage and is crucial for candidiasis [21], which is still predominantly caused by *C. albicans* [19].

*C. albicans* and *S. aureus* have been co-isolated from biofilm-associated diseases such as stomatitis, periodontitis, and also burn infections [22], which can worsen the cause of the disease compared to an infection with either pathogen alone [22,23].

Besides that, infected wounds carry an increased risk of bloodstream infection [24]; thus, there could be not only a risk of an interkingdom wound biofilm but also a risk of polymicrobial bloodstream infection, which highlights the importance of early treatment.

Current research on the management of interkingdom biofilms, such as *C. albicans*–*S. aureus* biofilms, as well as evaluation of their tolerance, has primarily focused on antibiotics/antimycotics [23,25]. However, when treating (chronic) wounds without systemic infection, the use of antibiotics should be carefully evaluated to prevent bacteria from building antibiotic resistance [24]. On the one hand, many patients with chronic wounds suffer from poor vascularization due to PAD or diabetic macro- and microangiopathy. This results in systemically administered antibiotics reaching only a comparatively low serum concentration, particularly in the peripheral areas of wounds on the lower legs and feet. On the other hand, antiseptics like octenidine dihydrochloride, polyhexamethylene biguanide or iodine are superior to antibiotics in the local and topical treatment of chronic wounds since they are, by definition, “broad-spectrum agents” that are effective not only against (a class of) bacteria but also against fungi and viruses [26]. However, to be effective, they should be combined with wound debridement. The importance of systemic antibiotics for systemic or deep tissue infections remains beyond dispute [27].

In the typical scenario, antiseptics are tested under standardized test conditions like DIN EN 13727 that do not adequately reflect clinical practice [28,29,30]. As a purely laboratory-based suspension test, it evaluates microorganisms in free liquid under standardized conditions; in living tissue, however, complex biological barriers, biofilms, protein contamination, and interactions with living cells are prevalent.

For this reason, translational human-like models can provide a more detailed understanding of wound microbiology and tolerance to antimicrobial agents in biofilms. To address the lack of research on fungal and interkingdom biofilms in chronic wounds, this study investigates the response of *C. albicans* to clinically relevant antiseptics, both alone and in combination with *S. aureus*, in a leucocyte-rich human plasma biofilm model (lhBIOM).

## 2. Results

The focus of this study was to evaluate the interaction among different microbial species within a wound biofilm model.

### 2.1. Comparison of Scanning Electron Microscopy Images from lhBIOM and Patients’ Slough

**Figure 1:**
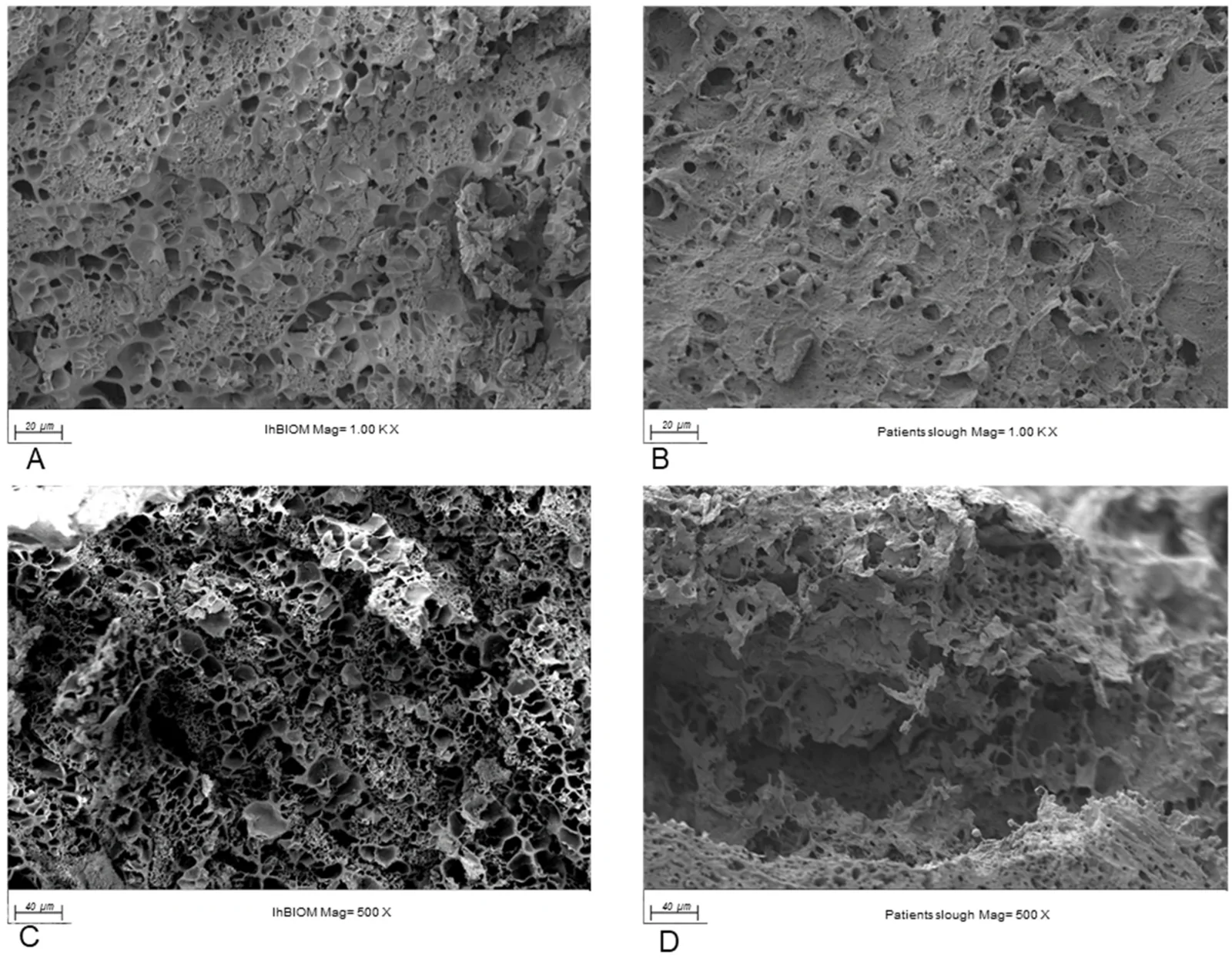
Sample scanning electron microscopy images of leucocyte-rich human plasma biofilm model (lhBIOM) (**A**,**C**) and patient slough (**B**,**D**) for a visual comparison of lhBIOM structures with actual wound environment.

When comparing electron microscope images of the patient’s wound slough and the human lhBIOM at 500× and 1000× magnification, the matrix looked quite similar in both (Figure 1A–D). This applied not only to the surface structure but also to the crevices where the microorganisms could settle. Both samples also exhibit an uneven distribution of microorganisms, which appeared as scattered clusters.

**Figure 2:**
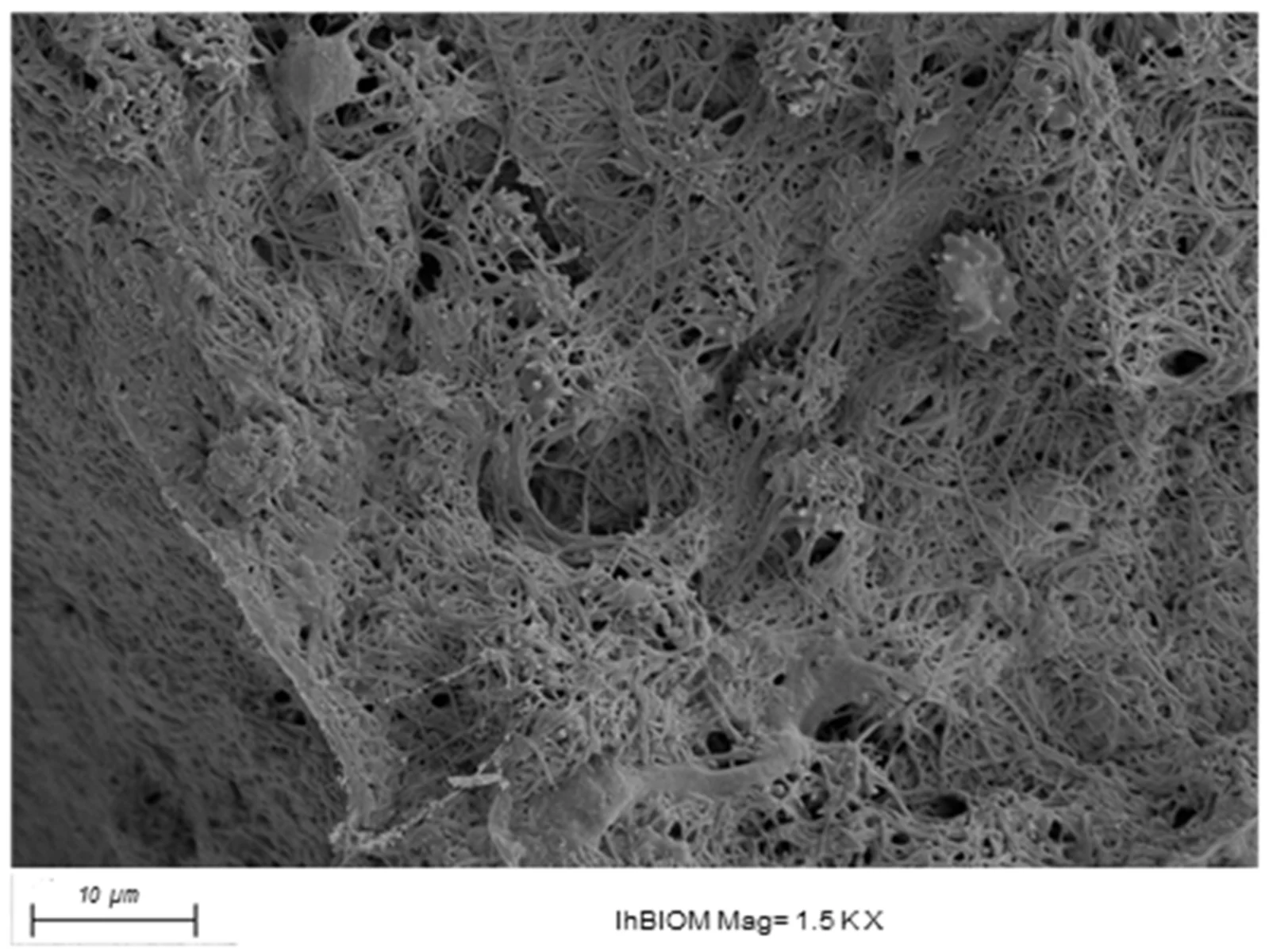
Sample scanning electron microscopy images of immune cells in a leucocyte-rich human plasma biofilm model (lhBIOM).

Furthermore, immune cells from the blood donor are also visible in isolated cases (Figure 2). This is yet another parameter demonstrating the comparability of the lhBIOM, which distinguishes it from other 2D and 3D biofilm models.

**Figure 3:**
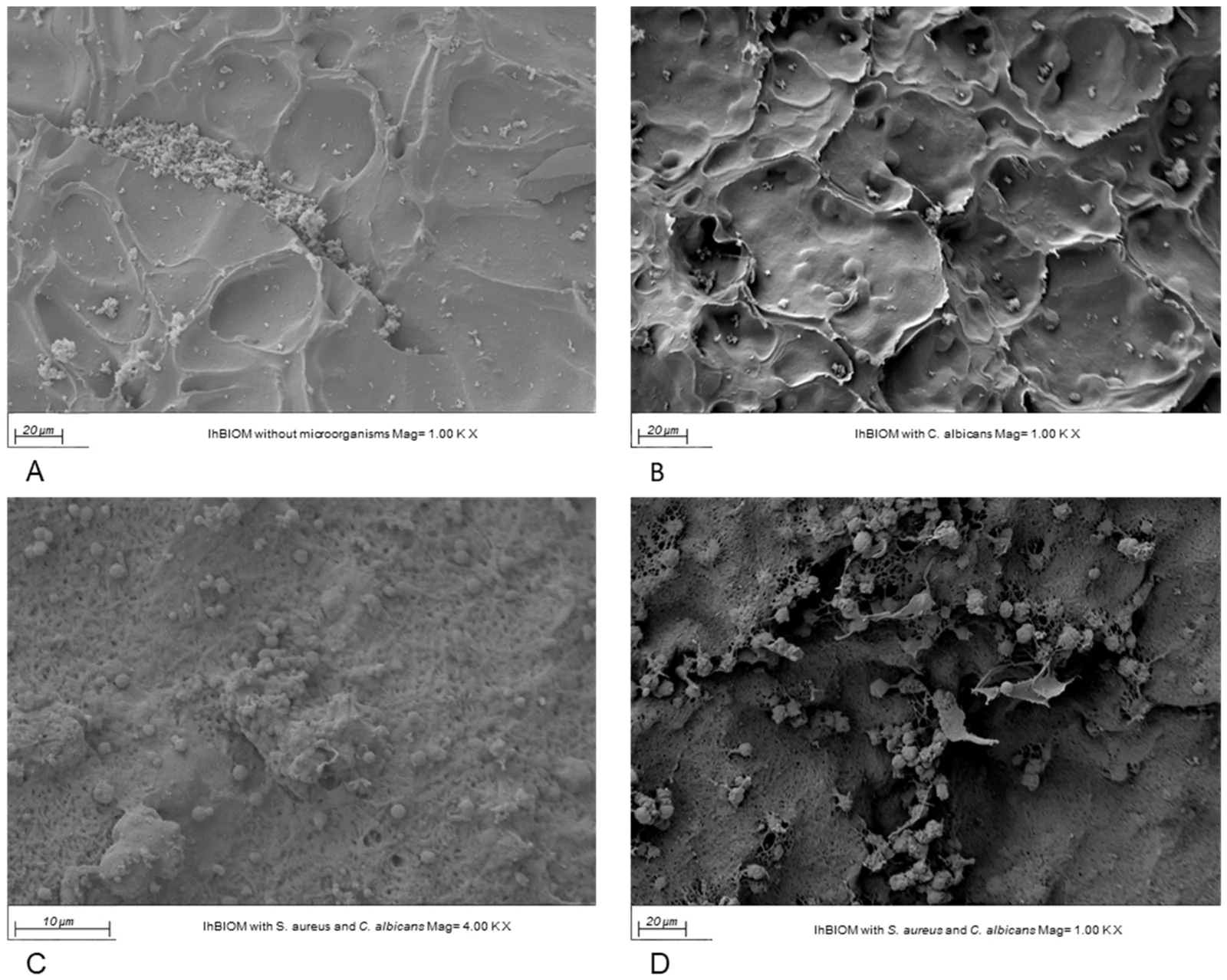
Sample scanning electron microscopy images of a leucocyte-rich human plasma biofilm model (lhBIOM) to compare models without microorganisms (**A**), *C. albicans* (**B**), as well as the combination of *S. aureus* and *C. albicans* (**C**,**D**).

When comparing inoculated with uninoculated lhBIOMs, the electron microscopic surface structure of the model without microorganisms appeared relatively smooth (Figure 3A). In contrast, models containing inoculum exhibited a coarser surface, for example due to septation resulting in the formation of chamber-like structures (Figure 3B). In the combined model, both *S. aureus* and *C. albicans* could be observed (Figure 3C). However, at a low magnification, it was difficult to distinguish between *C. albicans* and *S. aureus* grape-like clusters (Figure 3D), such that, at least under the electron microscope, no competition with the superiority of one of the two microorganisms is detectable. Different morphologies of *C. albicans* could also not be clearly discernible.

### 2.2. Evaluation of Antiseptic Efficacy in Mono- and Duo-Species Wound Biofilms

In the lhBIOM, *S. aureus* and *C. albicans* can be examined in single-species and interkingdom biofilms in a wound-like environment containing human immune cells. Using a quantitative suspension method, the effectiveness of the treatment with antiseptics was quantified, and differences in growth between the monocultures of *S. aureus* and *C. albicans* and the mixed culture were analyzed. The first treatment of the lhBIOM was administered after a 24 h incubation period, regardless of whether the models were inoculated with one or both species.

**Figure 4:**
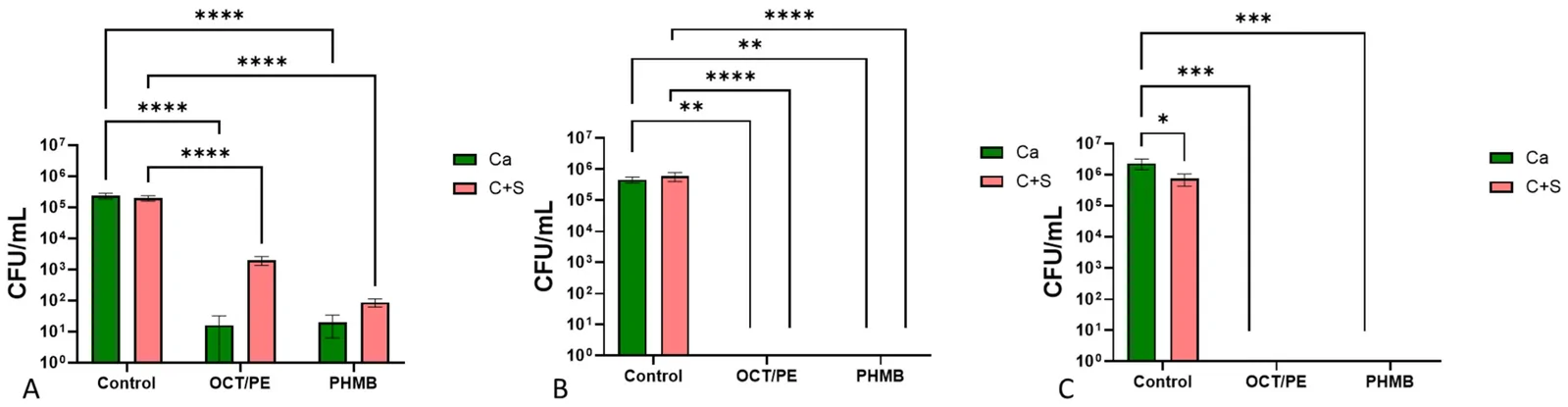
Microbial count of *C. albicans* as a single-species culture (Ca) and in co-cultivation with *S. aureus* (C + S) after treatment with octenidine-dihydrochloride/phenoxyethanol (OCT/PE), polyhexamethylene biguanide (PHMB) and the control after 24 (**A**), 48 (**B**) and 72 h (**C**). (Values as mean ± SEM; * *p* < 0.05 ** *p* < 0.01 *** *p* < 0.001 **** *p* < 0.0001 n = 3.)

Using antibiotic plates, the survival of *C. albicans* was evaluated. As shown in Figure 4, *C. albicans* was significantly reduced following treatment with the tested antiseptics.

There was also a tendency for the *C. albicans*–*S. aureus* co-culture to exhibit greater tolerance to OCT/PE and PHMB than *C. albicans* alone. However, 72 h after treatment (96 h after co-cultivation), *C. albicans* showed a significant decrease (Ca 2.3 × 10^6^ CFU/mL vs. C + S 7.4 × 10^5^ CFU/mL) in growth with *S. aureus* in the control. Apart from that, 48 h after antiseptic treatment, no *C. albicans* CFU could be detected in either model (Figure 4).

**Figure 5:**
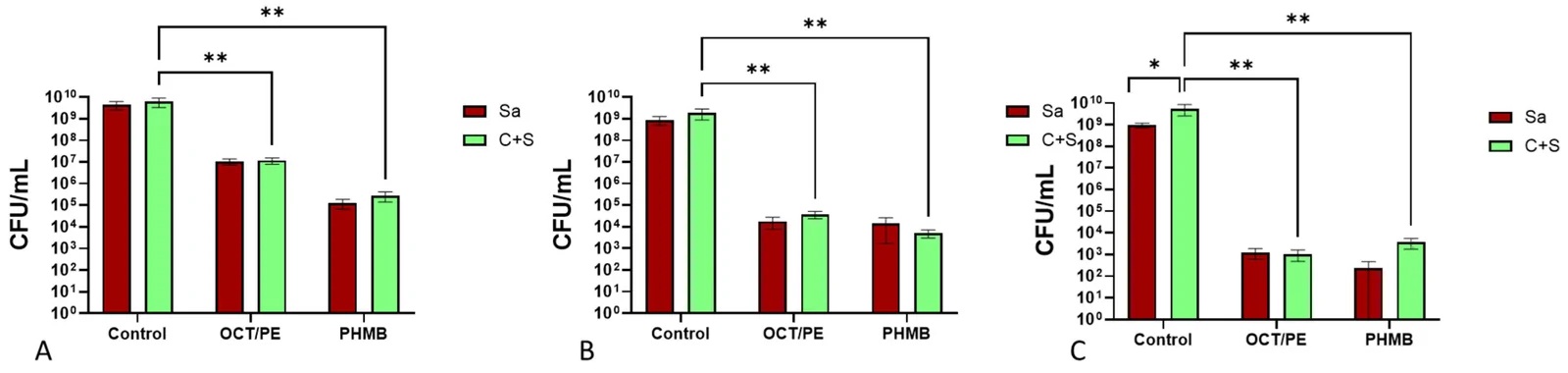
Microbial count of *S. aureus* as single-species culture (Sa) and in co-cultivation with *C. albicans* (C + S) after treatment with octenidine-dihydrochloride/phenoxyethanol (OCT/PE), polyhexamethylene biguanide (PHMB) and the control after 24 (**A**), 48 (**B**) and 72 h (**C**). (Values as mean ± SEM; * *p* ≤ 0.05 ** *p* < 0.001 n = 3.)

In contrast, *S. aureus* showed a steady decline over time. However, it was not completely eradicated and remained detectable on antimycotic plates after 72 h (approx. 1 × 10^3^ CFU/mL), both in single-species and co-culture models (Figure 5). Furthermore, a significant difference in CFU/mL between the mono- and co-culture models was observed after 96 h of co-cultivation. Here, the number of CFU/mL was approximately five times higher than in the single-species sample (Figure 5).

No significant differences were observed among the antiseptics tested. However, a trend was noted suggesting that PHMB may be more effective against *C. albicans* and *S. aureus* than OCT/PE after 24 h (Figure 4 and Figure 5).

### 2.3. Confocal Laser-Scanning Microscopy of Interkingdom Biofilms

**Figure 6:**
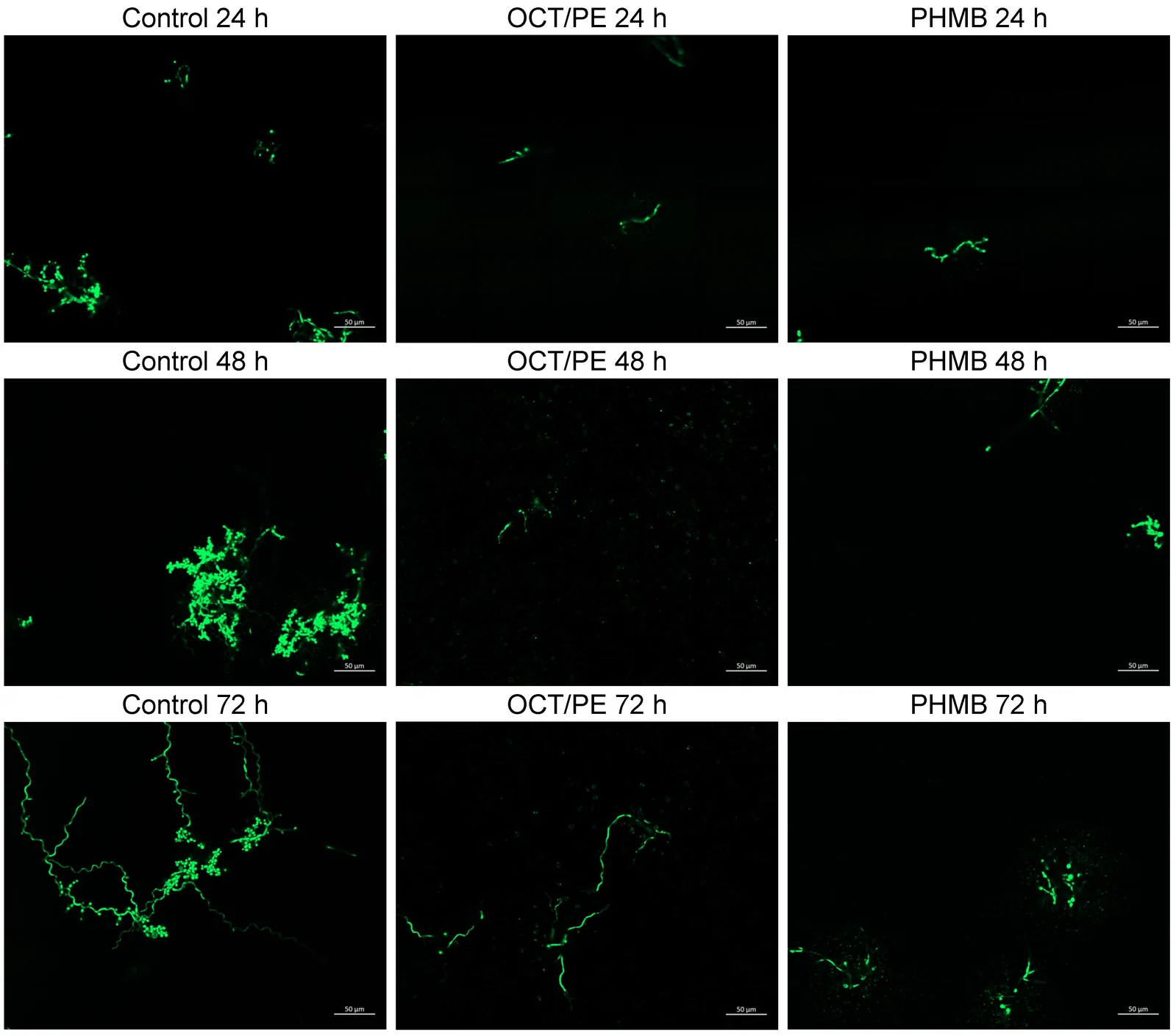
Confocal laser-scanning microscopy mosaic images consisting of 20 single images of lhBIOM with *C. albicans* (green; sfGFP) in single-species culture with control, octenidine-dihydrochloride/phenoxyethanol (OCT/PE) or polyhexamethylene biguanide (PHMB) (63× magnification) (n = 3).

**Figure 7:**
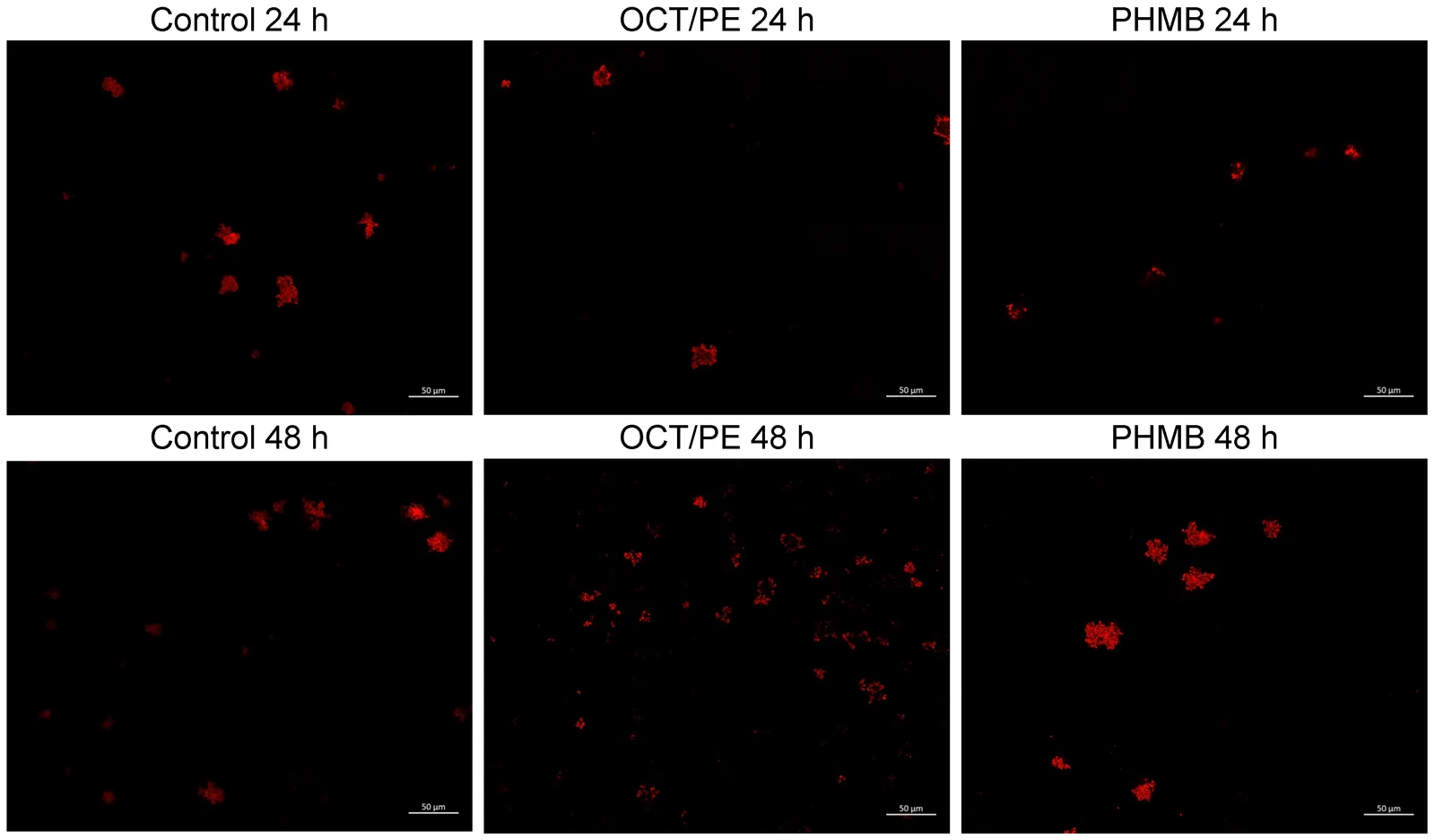

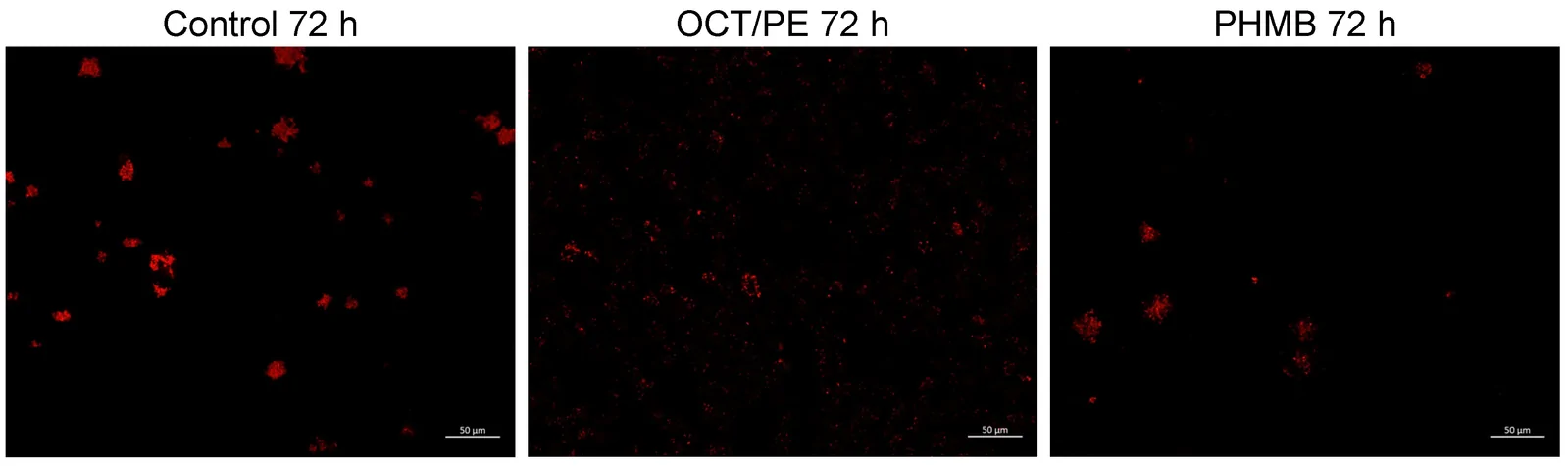
Confocal laser-scanning microscopy mosaic images consisting of 20 single images of lhBIOM with *S. aureus* (red; mCherry) in single-species culture with control, octenidine-dihydrochloride/phenoxyethanol (OCT/PE) or polyhexamethylene biguanide (PHMB) (63× magnification) (n = 3).

**Figure 8:**
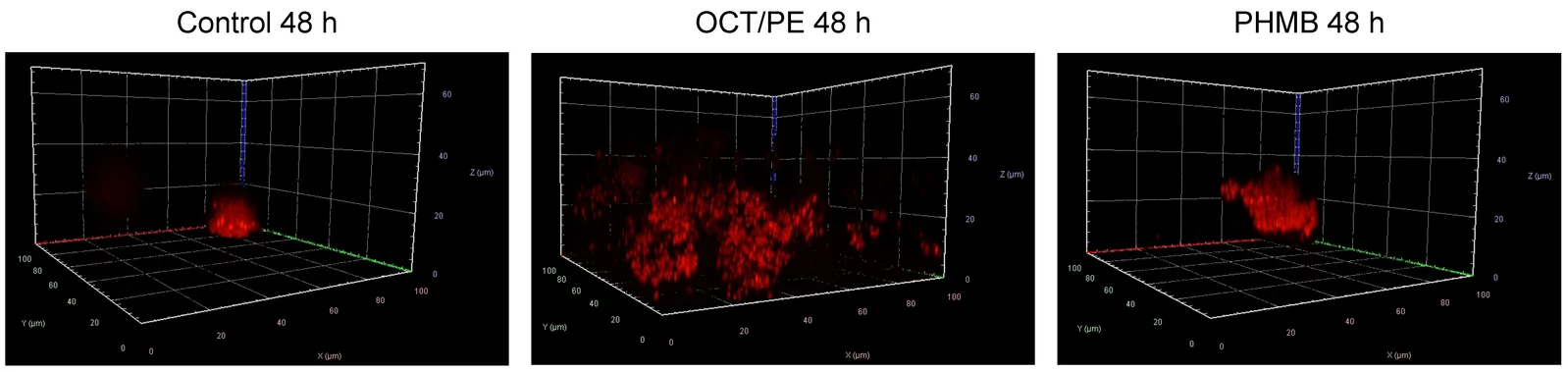
Confocal laser-scanning microscopy 3D images consisting of lhBIOM with *S. aureus* (red; mCherry) in single-species culture with control, octenidine-dihydrochloride/phenoxyethanol (OCT/PE) or polyhexamethylene biguanide (PHMB) 48 h after treatment (63× magnification) (n = 3).

Using fluorescently labeled *S. aureus* (SH1000; mCherry) and *C. albicans* (SC5314; sfGFP), the spatial distribution and morphology were observed. Regardless of culture and treatment conditions, the microorganisms were not evenly distributed, as shown in Figure 6, Figure 7 and Figure 8. Several clusters with higher microbial abundance were observed.

#### 2.3.1. *C. albicans* Single-Species Biofilm

In the laser-scanning images, the spatial distribution and individual morphology were observable.

The *C. albicans* monoculture appeared to exhibit a decrease in cell count between the samples treated with OCT/PE or PHMB and the control, as expected from the previous experiments. While *C. albicans* in the single-species culture control group seemed more clustered, the treated samples were characterized by smaller and more isolated filamentous structures. Upon closer inspection of the cluster, both filamentous and cell forms were observed (Figure 6). Following treatment with PHMB, the filamentation was smaller and branched. Based on the morphology, it appeared that the treatment with antiseptics had led to the formation of pseudohyphae predominating over true hyphae.

Over the course of the observation period, it appeared that the branched and thickened areas in the control group became less prominent, whereas more uniform hyphae became visible. In most cases, filament formation followed a sinusoidal pattern and extended progressively away from the center (Figure 6).

#### 2.3.2. *S. aureus* Single-Species Biofilm

*S. aureus* showed a typical grape-like cluster, also known as a staphylococcal cluster, regardless of treatment and incubation time. There was no noticeable difference in terms of shape; only after OCT/PE treatment did *S. aureus* appear somewhat more diffuse (Figure 7).

However, analysis of the 3D images showed that, in OCT/PE-treated samples, *S. aureus* clusters became visible particularly in the lower section and had accumulated in large quantities (Figure 8).

#### 2.3.3. *C. albicans* and *S. aureus* Co-Biofilm

**Figure 9:**
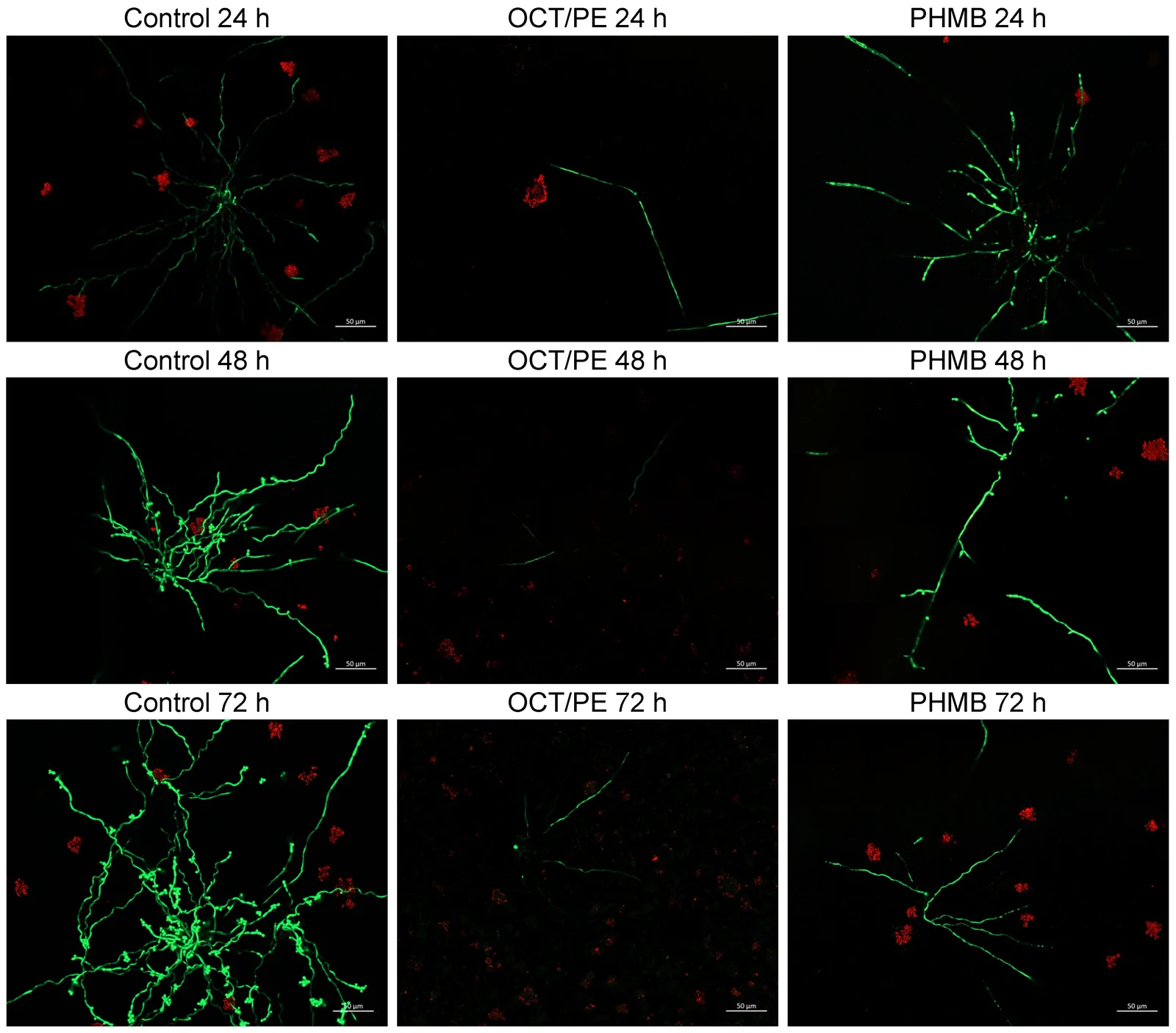
Confocal laser-scanning microscopy mosaic images consisting of 20 single images of lhBIOM with *C. albicans* (green; sfGFP) and *S. aureus* (red; mCherry) in co-culture with control, octenidine-dihydrochloride/phenoxyethanol (OCT/PE) or polyhexamethylene biguanide (PHMB) (time after treatment) (63× magnification) (n = 3).

The morphology of *C. albicans* in the co-culture with *S. aureus* differed markedly from that observed in monoculture. In contrast to the single-species culture (Figure 6), cell morphology was less distinct. Instead, *C. albicans* exhibited extensive sprawling filamentation in co-culture. The hyphae were not confined to the central region, but spread outward from the center (Figure 9). This was observed regardless of treatment, although the overall incidence declined following the antimicrobial treatment. However, yeast cell structure became more visible in higher layers, where *C. albicans* accumulated and/or biomass seemed to pile up. Furthermore, the filamentation of antimicrobial-treated *C. albicans* seemed longer than in the single-species culture (Figure 6). In the control, 72 h after treatment and 96 h of co-cultivation, both sinusoidal and straight hyphae were observed, as well as cellular morphology. However, the latter, in particular, was present in areas where *C. albicans* occurred frequently (Figure 9).

**Figure 10:**
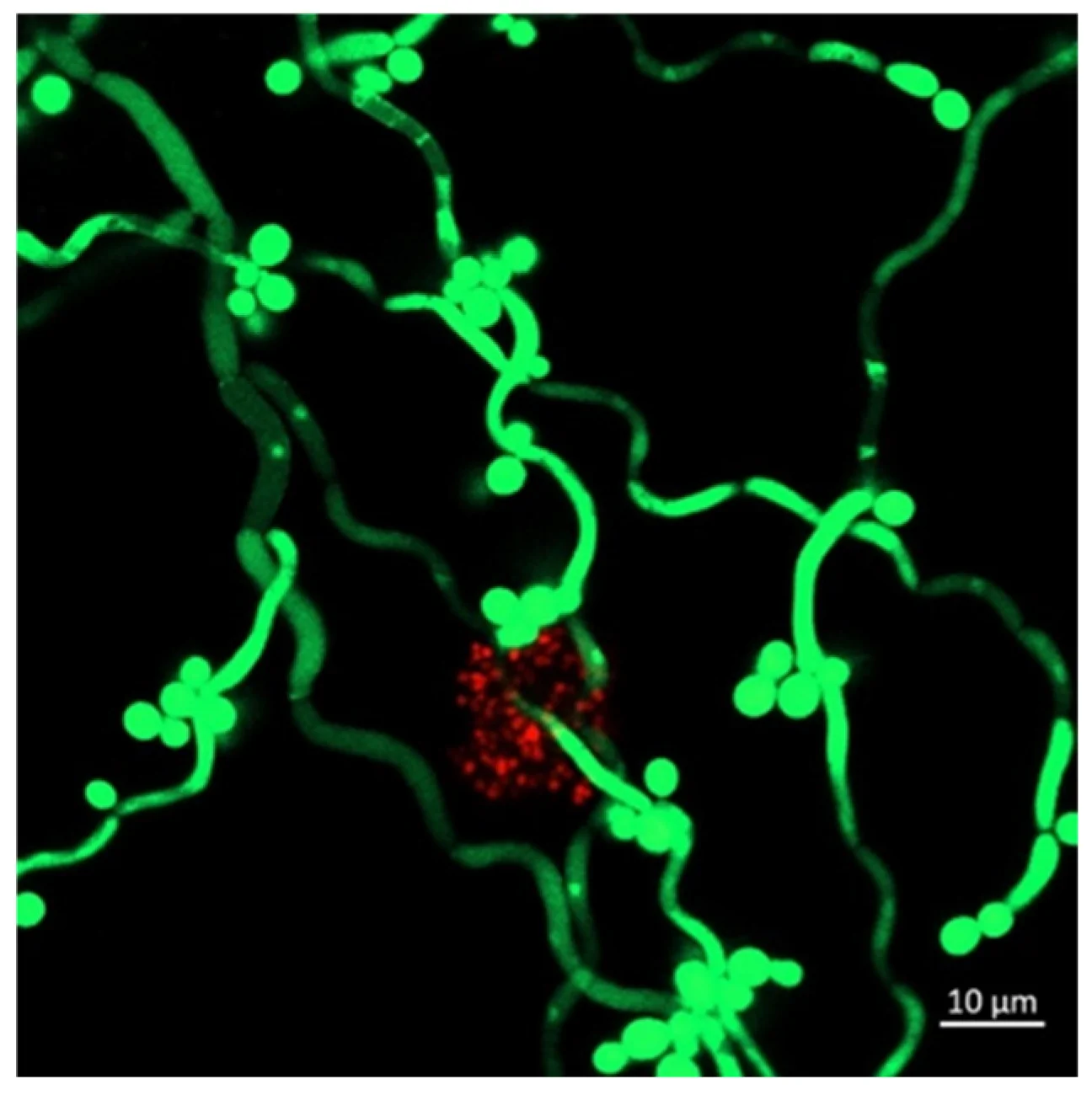
Confocal laser-scanning microscopy 3D images of lhBIOM with *C. albicans* (green; sfGFP) and *S. aureus* (red; mCherry) in co-culture 72 h after treatment.

*S. aureus* showed no noticeable changes in the shape of the clusters it formed; only after treatment with OCT/PE did the clusters appear more diffuse, similar to those in the single-species culture. However, they could be detected in proximity to the hyphae or seemed to be adhering directly to them, here, less in the center than in the periphery (Figure 9 and Figure 10). In particular, Figure 10 shows how *S. aureus* appears to be partially embedded between the hyphae of *C. albicans*.

## 3. Discussion

To treat infected, chronic wounds, it is necessary to study and understand the interactions among the organisms involved. Bacteria and bacterial biofilms play a major role in delayed healing of (chronic) wounds, but fungi should not be underestimated [13]. Therapeutic approaches that primarily target the bacterial burden (e.g., antibiotics) may promote fungal colonization and the risk of infection [12,31,32,33]. In addition, fungi involved in infection of hard-to-heal wounds can help bacteria resist antibiotics as well as the host’s immune response [4].

This brings the use of non-species-specific antimicrobial therapy for chronic wounds without systemic infection into focus [24]. This study aimed to investigate the effect of antiseptics in an interkingdom wound biofilm. The focus was on quantifying their efficacy against the microorganisms and the visualization of a possible interaction between the fungi and bacteria used. Furthermore, a leucocyte-rich human plasma biofilm model (lhBIOM) was used to simulate the wound environment. Although there is a wide variety of models that are used to study biofilms, the term “biofilm model” is inadequately defined and encompasses everything from one-dimensional in vitro models on microscope slides, steel, or plastic to cell cultures and models on animal skin [34,35,36]. In many of these models, the transition of the planktonic bacteria used into the biofilm state is induced by appropriate culture media. The influence of human material or even the factors of a clinical wound situation are not reflected in the results [37]. However, there are also more complex models that observe bacterial growth in a human environment [38,39,40]; the lhBIOM used here also belongs to this category. Electron microscopic comparison with real human biofilms on wounds reveals structural parallels, and the distribution and embedding of the bacteria or fungi are quite similar (Figure 1). In this study, from patients with high levels of slough and biofilm formation, a three-dimensional coating—often up to 2 mm thick and resembling a carpet—was collected and analyzed using SEM. However, when examining the structural similarities and differences between the biofilms of *C. albicans* and *S. aureus*, it was difficult to identify morphological and qualitative differences between the two organisms (Figure 3). For this reason, it requires other methods, such as LSM or—from a quantitative perspective—QSM, which were used in this study.

The results show that *C. albicans* may benefit from the presence of *S. aureus* after 24 h of treatment; more yeast colonies were detectable in the combined biofilm models than in the treated monocultures. After two applications of the antiseptics, no yeast colonies were detectable in either. This influence could not be confirmed on the part of *S. aureus* in co-culture, although this has been described under the influence of antibiotic treatment [23]. Kong et al. showed a significant advantage for *S. aureus* in polymicrobial biofilms with *C. albicans* against Vancomycin. They assume that the matrix, or the matrix components, have a positive effect on *S. aureus* [41]. In the current experimental setup, no colony-forming units of *C. albicans* were detected 48 h after treatment (72 h of co-cultivation) but, because of the CLSM images, fragments of presumably dead cells could be seen. Nevertheless, no quantitative differences were observed for *S. aureus* with fragments of dead *C. albicans*, extracellular substances, or the live *C. albicans* that could be analyzed within 24 h of treatment.

However, it appears that *S. aureus* gained an advantage in the control group, due to the presence of *C. albicans*, in contrast to the single-species culture. In this case, more CFU/mL of *S. aureus* were detected compared to the monoculture. Krause et al. observed a stimulative influence of prostaglandin E_2_ on the growth of *S. aureus* biofilms with a remarkable increase after 72 h [42]. Similar to that, we observed a slight increase in the CFU/mL at the outset of our investigation with the agent-free treatment, which became significant after 96 h of co-cultivation. At the same time, growth of *C. albicans* seems to be limited in the control group. This could be due to a lack of resources within the biofilm caused by the growth of *S. aureus*, which could limit the microbial fungus.

Besides that, *C. albicans* could promote the spread of *S. aureus* infections, as *S. aureus* appears to attach itself to the hyphae of *C. albicans*. In this way, *S. aureus* could reach deeper tissue layers and additional areas [43]. From this perspective, it becomes clear how important it is to monitor fungal colonization in (chronic) wounds. Not only can the infection and the molecular promotion of bacterial growth be an ongoing problem, but hyphal formation can also penetrate tissue [15]. In this way, a local infection can spread and lead to a systemic infection as *S. aureus*—attached to the hyphae—penetrates deep tissue and enters the bloodstream [43].

Apart from that, *C. albicans* is known for its filamentation. In the past, several causes for the filamentation have been identified, for example, a temperature of about 37 °C, a neutral to alkali pH value, serum, and a high concentration of carbon dioxide [44,45]. Based on the experimental setup, it seems likely that *C. albicans* forms filaments from the beginning, because the yeast was incubated at 37 °C in blood/plasma, which typically has a neutral to slightly alkaline pH (pH 7.35–7.45).

In general, the transition from yeast to hyphae is inhibited once cell density reaches a certain level due to the accumulation of the quorum sensing molecule farnesol [15,45]. Yeast cells could be observed primarily in the center of the cluster, respectively, on spots where *C. albicans* appeared in piles, which gives rise to the assumption that the farnesol production may be locally increased in the wound clot model, but this contradicts the fact that there was an increased filamentation over time in the single-species culture. This may be because, while farnesol prevents yeast cells from transitioning into hyphae, it is unable to inhibit the elongation of hyphae that have already formed [45].

In particular, increased filamentation in co-culture with *S. aureus* became clearly visible. Several publications point out that bacterial peptidoglycan is one of the most potent inducers for filamentation [33,46,47]. Tan et al. reported an increased release of peptidoglycan following the administration of beta-lactam antibiotics, leading to filamentation of *C. albicans*, in tissue niches where yeast morphology actually occurs. This can enhance the virulence of *C. albicans* and facilitate its penetration into tissue [33]. Furthermore, similar molecules have been detected in human serum; it is assumed that these also originate from bacteria in the gut microbiome [46]. Perhaps that could explain the increased hyphae formation in combination with *S. aureus* (Figure 7) but not the observations in the single-species culture shown here (Figure 6).

Hopke et al. observed that neutrophil swarming resulted in delayed germination [48]. It seems most likely that the immune competence of the leucocyte-rich human biofilm model limited hyphae formation and, over time, the immune cells starved, and more filamentation could be visible. In addition, in co-culture, *S. aureus* could limit the influence of the immune cells [49], so that not only could the bacterial components accelerate hyphae growth but inhibitory factors would also be mitigated. At the same time, this could be a reason for the trend observed in *C. albicans* toward increased tolerance to the tested antiseptics. Because of the host’s reduced immune response, there is one less aggressor.

Apart from that, Fang et al. described that octenidine-dihydrochloride can inhibit filamentation of *C. albicans* [50]. This effect was not consistently visible in the present model. Filamentous growth was particularly predominant in co-culture with *S. aureus*. However, in contrast to Fang et al., the lhBIOM contains a lot of filament triggers and, in addition, conventional clinical antiseptic solutions were used. For this reason, octenidine-dihydrochloride may limit hyphae formation but, in co-culture, the trigger for hyphae formation could predominate. Nevertheless, the eradicating effect of both antiseptics against *C. albicans* was shown.

This translational study once more highlights the importance of non-species-specific antimicrobial treatment. Treatment with OCT/PE and PHMB was effective against *C. albicans* and substantially reduced *S. aureus*, although the bacterium was not eliminated after 72 h. These results show that the tested antiseptics are effective against both microorganisms, but that is not yet sufficient for the *S. aureus* biofilm. In everyday clinical practice, debridement is the method of choice. However, this method requires time, money, and, not least, the necessary know-how [51,52]. For this reason, it is still necessary to find a combined solution to combat biofilm and the microorganisms embedded within it. To achieve this goal, further research is needed to identify synergies and effects against antimicrobial treatment, including those related to interkingdom biofilms.

## 4. Methods

### 4.1. Preparation of an Interkingdom Leucocyte-Rich Human-Plasma Biofilm Model (lhBIOM)

To mimic the wound environment, a biofilm model of human plasma, immune cells, and pathogens was generated as previously described [53]. Freshly collected leucocyte accel from thrombocyte donation of the transfusion medicine (University Hospital Hamburg-Eppendorf, Hamburg, Germany) was centrifuged for 30 min at 1600× *g* and room temperature in a sterile centrifuge tube (Eppendorf centrifuge 5804R; Hamburg, Germany) to separate the content of the accel into three phases. The plasma and buffy coat layers were collected, while allowing minimal contamination from the erythrocyte layer, and mixed with one bag of fresh frozen AB plasma (Transfusion medicine University Hospital Hamburg-Eppendorf, Hamburg, Germany).

Afterwards, the microbial strains *Staphylococcus aureus* (SH1000) and *Candida albicans* (DSM 1386) were added. Therefore, the strains were pre-cultured overnight in casein/soy peptone broth (CSB 15 g/L casein peptone, 5 g/L soy peptone, and 5 g/L NaCl), then adjusted to an optical density of 0.1 at a wavelength of 600 nm (OD_600_) before use; these precultures were diluted 1:100 with the plasma immune cell mixture and, for coagulation models, 18.3 µL 0.5 M calcium chloride per milliliter and pipetted promptly in 12-well plates (1.5 mL per well).

### 4.2. Preparation for Scanning Electron Microscopy (SEM)

To demonstrate the validity and comparative significance of the human biofilm in relation to patients’ wound biofilm, scanning electron microscopy (SEM) images were generated. Patients with high bacterial loads on their wounds, which led to significant slough and biofilm formation, were selected for the collection of clinical wound biofilm. Ethical approval has been obtained for the collection and analysis of patients’ biofilm during standard medical care (PV5883-4183-BO-ff; last amendment 29 August 2024); patients provided their consent accordingly. For SEM imaging, patients’ wound biofilm and the pure models with *S. aureus* (SH1000; Sa) and/or *C. albicans* (DSMZ 1386; Ca) (single and dual: C + S) and without microorganisms were prepared as described above and incubated for 48 h without any treatment.

Both lhBIOM and wound slough were fixed in glutaraldehyde/PVP solution (20% Polyvinylpyrrolidone, 0.52% sodium nitrate, and 2.5% glutaraldehyde in 0.1 M cacodylate buffer) for one hour at 4 °C. Subsequently, the samples were washed three times with 0.1 M cacodylate buffer and cryofixed using nitrogen and frozen at −20 °C until further processing. The glycocalyx preparation was carried out with arginine HCl solution (2% arginine HCl, 2% glycine, 2% sucrose, and 2% sodium glutamate) for 18 h at room temperature. After a second wash step (3 times aqua dest.), the samples were incubated for 5.5 h in tannic acid solution (2% tannic acid and 2% guanidine HCl). In conclusion, the samples were washed and stored in 0.1 M cacodylate buffer overnight (4 °C) and fixed for 30 min in 1% osmium tetroxide solution and washed and stored again in 0.1 M cacodylate buffer. The samples were dehydrated in decreased concentration of isopropanol (50%; 70%; 90%; 100% in aqua dest., 15 min each) and acetone (50%; 75%; 100% in isopropanol, 15 min each). Finally, models were critically dehydrated (Critical Point Dryer CPD 030, BAL-TEC AG, Balzers, Liechtenstein) and sputtered with gold (Cressington 108auto, Cressington Scientific Instruments Ltd., Watford, UK) before examining under the scanning electron microscope (SEM, Crossbeam 340, Zeiss AG, Oberkochen, Germany).

### 4.3. Quantitative Suspension Method (QSM)

For the quantitative suspension method (QSM), lhBIOM for single-species and dual-species models were prepared as previously described, whereas, in the latter case, both strains were combined 1:1 based on optical density (OD_600_). Accordingly, in dual-species cultures, the same inoculum volume (OD600 0.1 1:100 in lhBIOM) of each species as in the corresponding single-species cultures was used, ensuring an identical absolute inoculum size per species across all conditions.

Wound biofilm models were incubated for 24 h at 37 °C with shaking at 50 rpm. The antiseptics octenidine dihydrochloride/phenoxyethanol (0.1% OCT/2% PE; Octenisept Schülke&Mayr; Norderstedt, Germany) and polyhexamethylene biguanide (0.04% PHMB; Serasept 2 Serag-Wiessner; Naila, Germany) were tested in concentrations commonly used in clinical practice. These two antiseptics were selected because the additives in their clinically approved solutions have different potentials for disrupting the biofilm matrix and, accordingly, different levels of bactericidal activity within the biofilm are expected over time. For each 1.5 mL wound biofilm model, 300 µL antiseptic was pipetted onto the model, ensuring the entire surface was wetted. As a control, phosphate-buffered saline (PBS without Ca/Mg) was used. The models were treated 24, 48, and 72 h after inoculation of the models and incubated at 37 °C.

For analysis, the antiseptics remained on the models (for 24 h, 48 h, or 72 h) until they were neutralized with a standard neutralization solution (TLSNt-SDS: 6% polysorbate 80, 6% saponin, 0.8% lecithin, 2% sodium dodecyl sulfate, and 0.6% sodium thiosulfate in aqua dest.) for 5 min. The models were then dissolved with 1.5 mL of a bromelain solution (49.68 units/mL bromelain in 1× PBS) for 2 h.

After that, liquefied extracts were serially diluted and plated onto selection plates (CSA 15 g/L casein peptone, 5 g/L soy peptone, 5 g/L NaCl, 15 g/L Agar) to distinguish between *S. aureus* (antimycotic 25 µg/mL nystatin dihydrate) and *C. albicans* (antibiotic 60 µg/mL tetracycline and 30 µg/mL chloramphenicol) and incubated overnight at 37 °C. Colonies were counted by a colony counter Scan 500 (interscience; Saint-Nom la Bretèche, France).

### 4.4. Visualization of the Interkingdom Biofilm by Using Confocal Laser-Scanning Microscopy (CLSM)

For the laser-scanning microscopy, *S. aureus* (SH1000) labeled with mCherry and *C. albicans* (SC5314) labeled with sfGFP were used [54]. For each microorganism, or in a 1:1 mixture (based on OD_600_), volume-matched lhBIOM models were prepared and placed directly into Ibidi chamber slides (Ibidi GmbH; Gräfelfing, Germany). As mentioned above, the models were incubated for 24 h before the treatment. In the case of the dual-species models, *S. aureus* and *C. albicans* had therefore already been growing in parallel for 24 h before the first treatment. Incubation took place in a humid environment, and they were treated every 24 h as described previously. The wound biofilm models in the chamber slides were examined using confocal laser-scanning-microscopy (Axio Observer. Z1/7 LSM 800 with Airyscan Carl Zeiss AG; Jena, Germany) and a C-Apochromat 63×/1.20 W Korr UV VisIR objective. Two types of images were generated: bottom mosaic images consisted of 20 single shots and z-Stackimages using the ZEN software (version 2.3; Carl Zeiss Microscopy GmbH; Hamburg, Germany). Each analysis was prepared from three different immune cell donors. Bright-field images were also taken as fluorescence images for sfGFP (λ 488 nm; λ Ex: 488/Em λ 509 nm) and mCherry (λ 561 nm; Ex λ: 587/Em λ 610 nm).

### 4.5. Statistical Analysis

All analyses were performed in at least 3 biological replicates (1 biological replicate = 1 donor of trima accel) with technical triplicates. Data were analyzed with GraphPadPrism 10 (GraphPad Software, Inc., La Jolla, CA, USA) and displayed as mean ± standard error of the mean (SEM). Statistical analysis was performed using two-way ANOVA, followed by the Holm-Šídák test for multiple comparisons.

## 5. Conclusions

Chronic wounds are primarily associated with biofilms, which are not necessarily of purely bacterial origin. Fungi such as *C. albicans* can influence the growth of the primary bacterial pathogen and thus reduce the chances of healing. To counteract these local infections on chronic wounds at an early stage, nonspecific antimicrobial treatment is necessary. Antiseptics such as octenidine dihydrochloride/phenoxyethanol and polyhexamethylene biguanide are effective against both fungi (*C. albicans*) and bacteria (*S. aureus*). However, repeat treatment is important to ensure effective infection management against both.

## Data Availability

The original contributions presented in this study are included in the article. Further inquiries can be directed to the corresponding authors.
